# AstraZeneca ChAdOx1-S COVID-19 vaccine can be safely administered in patients with EDTA allergy

**DOI:** 10.1186/s13223-022-00665-3

**Published:** 2022-03-10

**Authors:** Syed B. Ali, Griffith Perkins, Dongjae Ryoo, Maverick Lee, Matthew Tunbridge, Chino Yuson, William Smith, Pravin Hissaria, Thanh-Thao Le

**Affiliations:** 1 Department of Clinical Immunology and Allergy, Le. Royal Adelaide Hospital, 1 Port Road, Adelaide, South Australia 5000 Australia; 2 Department of Immunopathology, SA Pathology, Adelaide, Australia; 3 School of Biological Sciences, University of Adelaide, Adelaide, Australia

**Keywords:** COVID-19, Vaccine allergy, EDTA, Excipients

## Abstract

**Background:**

Immediate hypersensitivity reactions to COVID-19 vaccines have been postulated to be linked to their excipients, such as polyethylene glycol (PEG) in Pfizer Comirnaty, or polysorbate 80 and ethylenediaminetetracetic acid (EDTA) in AstraZeneca ChAdOx1-S [recombinant] (Vaxzevria). These excipients are found in a range of other products, including injectable and oral medications as well as intravenous radiocontrast media (RCM) and various cosmetic products.

Patients with proven excipient allergy may be advised to avoid a COVID-19 vaccine containing that excipient and/or potentially cross-reactive excipients. This may result in individual patients not receiving vaccines, especially if an alternate option is not available, and on a broader level contribute to vaccine hesitancy. We present two cases of previously confirmed EDTA anaphylaxis with positive intradermal testing, who had negative Vaxzevria vaccine in-*vivo* testing and subsequently tolerated the vaccine.

**Case 1:**

A patient with history of anaphylaxis to RCM and local anaesthetics (LA) had positive intradermal test (IDT) to EDTA nine years earlier. Skin testing to Vaxzeria vaccine (up to 1:10 IDT), Comirnaty vaccine (up to 1:10 IDT) and EDTA 0.3 mg/mL IDT were negative. However, following EDTA 3 mg/ml IDT, he developed immediate generalised urticaria without anaphylaxis. Basophil activation testing was negative to disodium EDTA, Vaxzevria and Cominarty vaccines. Given the negative in-*vitro* and in-*vivo* testing to Vaxzevria vaccine, he proceeded to Vaxzevria immunisation and tolerated both doses.

**Case 2:**

A patient with history of anaphylaxis to RCM had positive skin testing to EDTA and RCM containing EDTA six years earlier. Following referral to COVID19 vaccine clinic, Vaxzevria vaccine (1:10 IDT) and Cominarty vaccine (1:10 IDT) were negative whilst EDTA was positive at 0.3 mg/mL IDT. He subsequently tolerated both Vaxzevria vaccinations.

**Conclusion:**

Excipient allergy does not necessarily preclude a patient from receiving a vaccine containing that excipient. Allergy testing can help identify excipient-allergic patients who may still tolerate vaccination, which is important in situations where COVID-19 vaccination options are limited.

## Introduction

The rate of severe immediate allergic reactions following COVID-19 vaccines is estimated at 4.7 per 1,000,000[1]. Some of these reactions have been attributed to an underlying excipient allergy; namely polyethylene glycol (PEG) in Comirnaty and Moderna Spikevax mRNA-1273 vaccines, tromethamine in Moderna and polysorbate 80 and ethylenediaminetetracetic acid (EDTA) in Vaxzevria (ChAdOx1-S; AstraZeneca) adenovirus vector vaccine. Although skin testing to PEG and polysorbate 80 has been widely used,

methodology is not standardized with a variety of testing reagents employed, and positive and negative predictive values of testing are unknown. However, there is no data on EDTA, which has been implicated in both immediate or type 1 hypersensitivity reactions with radiocontrast media (RCM) and local anaesthetic (LA) [2] as well as type 4 hypersensitivity reactions [3]. This compound is present in two forms, calcium disodium EDTA (cdsEDTA) and non-chelated disodium EDTA (dsEDTA), and is used in various cosmetics and pharmaceuticals as preservatives and stabilisers [2]. We present two cases of confirmed EDTA anaphylaxis, who tolerated both doses of Vaxzevria.

## Case 1

A 66-year-old man was referred in 2014, following rapid-onset generalised urticaria after intravenous iopimadol (Isovue 370) radiocontrast media (RCM) in 2009. Three years later, he developed immediate palmoplantar pruritis, facial oedema and urticaria with subcutaneous lignocaine and adrenaline (Lignospan Special) local anaesthetic (LA).

A common excipient, dsEDTA, was noted between the Isovue 370 RCM and Lignospan Special LA. Intradermal testing (IDT) was positive to Isovue 370 and other EDTA-containing RCM (Optiray 240, Ultravist 370, Visipaque 320, Conray 280), but not iomeprol (Iomeron 300) RCM (1:10 dilution), which does not contain EDTA. IDT to cdsEDTA was strongly positive at 0.3 mg/mL. A subcutaneous EDTA challenge (0.1 mL) with the patient’s consent was performed and he developed mild flushing within 15 min but tryptase levels were normal. Lignocaine challenge (without additives) was negative. Basophil activation test (BAT) was positive to EDTA-containing RCM (iotalamic acid; Conray 280 at 1:10 and 1:100 dilutions), cdsEDTA and dsEDTA (0.1 mg/mL and 0.3 mg/mL).

He was re-referred in 2021 to the COVID-19 vaccination clinic. Since previous consultation, he had one relevant episode, with facial urticaria to topical shaving lotion containing EDTA. IDT to Vaxzevria (1:10 dilution), Comirnaty vaccine (1:10 dilution) and dsEDTA (0.3 mg/mL) were negative. However, within 5 min of dsEDTA 3 mg/ml IDT, he developed immediate generalised urticaria, which resolved with oral cetirizine 10 mg. A tryptase level drawn within an hour of symptom onset was normal (5.3 µg/L) and comparable to patient’s baseline tryptase level of 4.8 µg/L. BAT was negative to dsEDTA (0.1 mg/mL and 0.3 mg/mL), Vaxzevria (1:10) and Comirnaty (1:10). He proceeded to first Vaxzevria immunisation with no adverse reactions and tolerated second vaccination as a single dose.

## Case 2

A 77-year-old man was referred to the immunology unit in 2014. He received intravenous Isovue 300 in 2012 and within five minutes had cardiopulmonary arrest requiring CPR and adrenaline. An incident tryptase was not performed but a baseline tryptase level was normal. Skin prick testing to multiple EDTA-containing RCM (including Isovue), cdsEDTA and dsEDTA (0.3 mg/mL and 3 mg/ml) was positive. IDT and intravenous challenge to non-EDTA containing Iomeron RCM was negative.

He was re-referred in 2021 to the COVID-19 vaccination clinic, during the interval between first consult and re-referral he had had one episode of immediate generalised pruritis and erythema after using body wash containing EDTA. He had negative IDT to Vaxzevria (1:10), Comirnaty (1:10) and dsEDTA (0.3 mg/mL). However, dsEDTA IDT (3 mg/mL) was positive. BAT could not be interpreted as he was a non-responder. He proceeded to first Vaxzevria vaccination without any adverse effects and subsequently tolerated the second vaccine as a single dose.

## Discussion

Globally, over 5 billion COVID-19 vaccines have been administered and as such a non-trivial number of adverse reactions, including anaphylaxis, may be expected. Allergy protocols for those with a history suggestive of severe allergy to COVID-19 vaccine or its excipients quickly emerged with inclusion of PEG and polysorbate 80 skin testing [4, 5]. While Vaxzevria also contains EDTA, published test protocols do not contain reference to EDTA, possibly due to limited published reports of EDTA allergy.

To our knowledge, there has only been one case of immediate systemic reaction to EDTA published to date (case 1 of this article [2]). In contrast, EDTA contact dermatitis has been more frequently reported, highlighting its capacity as an immunoreactive molecule [3, 6].

In both of our cases, recent repeat EDTA skin testing elicited positive results, on a background of remote immediate systemic reaction to EDTA-containing injectable medication. However, allergy testing to Vaxzevria was negative and therefore the question arose whether to administer this vaccine to our patients, despite confirmation of ongoing EDTA allergy. An analogous situation was the previous concern regarding influenza vaccination in egg-allergic patients, which was subsequently proven to be safe due to the only miniscule quantities of ovalbumin in the vaccine [7]. We obtained informed consent from both our patients to administer Vaxzevria vaccine, which was tolerated with no adverse effects to both vaccines.

In Vaxzevria, the quantity of dsEDTA in each dose (0.5 mL) is 0.02 mg (Personal communication, AstraZeneca, 2021). This is lower than the amount used in EDTA IDT dose. For example, in case one, the eliciting dose for an immediate generalised cutaneous reaction was between 0.06 mg and 0.09 mg (IDT with 0.02–0.03 mL of 3 mg/mL solution). In contrast other vaccines such as varicella (Varivax), influenza quadrivalent (FluMist) and rabies (RabAvert), contain 0.3 mg of EDTA per dose [8, 9]. In our second case, the patient experienced severe anaphylaxis after receiving up to 100 mL of Isovue 300 RCM which contains 0.39 mg/mL of dsEDTA [10]. Similarly, in our first case, the patient received 5 mL of Lignospan which contains 0.25 mg/mL EDTA. Collectively, this raises possibility of a dose related mechanism and should increase confidence with the Vaxzevria vaccine considering the relatively low amount of EDTA present in the full dose.

Skin testing to other COVID-19 vaccine excipients has also shown a poor predictive value for subsequently tolerating the vaccine [11]. Specifically, in a cohort of 80 patients with reactions to either Comirnaty or Moderna mRNA vaccines, a majority (> 70%) of these patients tolerated the second dose independent of the skin test result to PEG and/or polysorbate 80 [11].

Although immediate adverse reactions to COVID-19 vaccines occur, typical IgE mechanisms to vaccine components may not be relevant. In our experience, immediate reactions following first COVID-19 vaccination commonly manifest as flushing, non-urticarial rash, subjective throat tightness and/or hypertension rather than the hypotension, urticaria and objective angioedema typically observed with IgE mediated allergy. Furthermore, in these patients a rise in tryptase has not been demonstrated and hence the consideration of alternative mechanisms such as complement activation-related pseudoallergy (CARPA) with a release of other mediators including leukotrienes, proteases and platelet activating factor should be considered [12, 13].

## Conclusion

Excipient allergy testing may not be useful for those with suspected allergic reactions to COVID-19 vaccines; conversely a history of excipient allergy does not necessarily preclude patient from receiving a vaccine containing that excipient. Important considerations include the nature and severity of the index excipient reaction and the amount of excipient present in the index drug compared to the vaccine. Allergy testing to vaccines can help identify excipient-allergic patients who may still tolerate immunisation especially where vaccination options are limited.

## Data Availability

Skin testing and BAT data, as well as further information on materials used in the investigations are available on request.

## Abbreviations

BAT: Basophil activation test
CARPA: Complement activation-related pseudoallergy
cdsEDTA: Calcium disodium EDTA
dsEDTA: Disodium EDTA
EDTA: Ethylenediaminetetracetic acid
IDT: Intradermal test
LA: Local anaesthetic
PEG: Polyethylene glycol
RCM: Radiocontrast media
